# Mitral valve replacement after transcatheter aortic valve implantation in a patient with rheumatic heart disease and prior Ross procedure: a case report

**DOI:** 10.1186/s43044-019-0030-2

**Published:** 2019-11-14

**Authors:** Khaled D. Algarni, Amr A. Arafat

**Affiliations:** 1 Department of Cardiac Science, King Saud University, Riyadh, Kingdom of Saudi Arabia; 2 Department of Adult Cardiac Surgery, Prince Sultan Cardiac Center, Riyadh, Kingdom of Saudi Arabia; 3 Department of Cardiothoracic Surgery, Tanta University, Tanta, Egypt

**Keywords:** Beating mitral valve replacement, Ross procedure, Transcatheter aortic valve implantation

## Abstract

**Background:**

Reoperations are required frequently after the Ross procedure in rheumatic patients. The use of transcatheter aortic valve implantation (TAVI) in those patients could decrease the risk of future open procedure; however, the outcome may be affected by the concomitant mitral valve disease, and subsequent mitral reoperation may distort the implanted aortic valve.

**Case presentation:**

We present a female patient who had a beating mitral valve replacement after valve-in-valve TAVI in a patient with prior Ross procedure. Weaning from cardiopulmonary bypass was difficult, and the patient needed extra-cardiac membrane oxygenation (ECMO) and intra-aortic balloon pump because of right ventricular dysfunction. The right ventricular dysfunction could be due to the concomitant coronary artery disease or air embolism during the beating mitral valve surgery. Recovery was gradual, and the patient was discharged after 33 days. Pre-discharge echocardiography showed a maximum gradient of 9 mmHg on the aortic valve and mild paravalvular leak.

**Conclusions:**

Mitral valve replacement in a patient with prior TAVI and the Ross procedure was feasible; it decreased the operative risk and did not distort the implanted aortic valve.

## Background

Despite the low valve-related complications achieved after the Ross procedure in the middle- and young-aged population compared to other prostheses, [1] the outcome is suboptimal in patients with rheumatic heart disease and re-intervention is common [2]. Currently, the indications of transcatheter aortic valve implantation (TAVI) have been expanded to include low-risk patients [3]; however, its application in young rheumatic patients has not been evaluated. The risk of complications after TAVI, including all-cause mortality and paravalvular leak, increased in patients with mitral valve disease, [4] and subsequent mitral valve surgery may affect the implanted aortic valve. The use of TAVI as an adjunctive procedure to surgery to decrease the operative risk has not been evaluated in the literature.

We present a case of rheumatic valve disease who presented after the Ross procedure with moderate aortic stenosis and severe regurgitation, double mitral lesion, and functional tricuspid regurgitation. The patient had valve-in-valve TAVI and beating mitral valve replacement.

## Case presentation

**Fig. 1:**
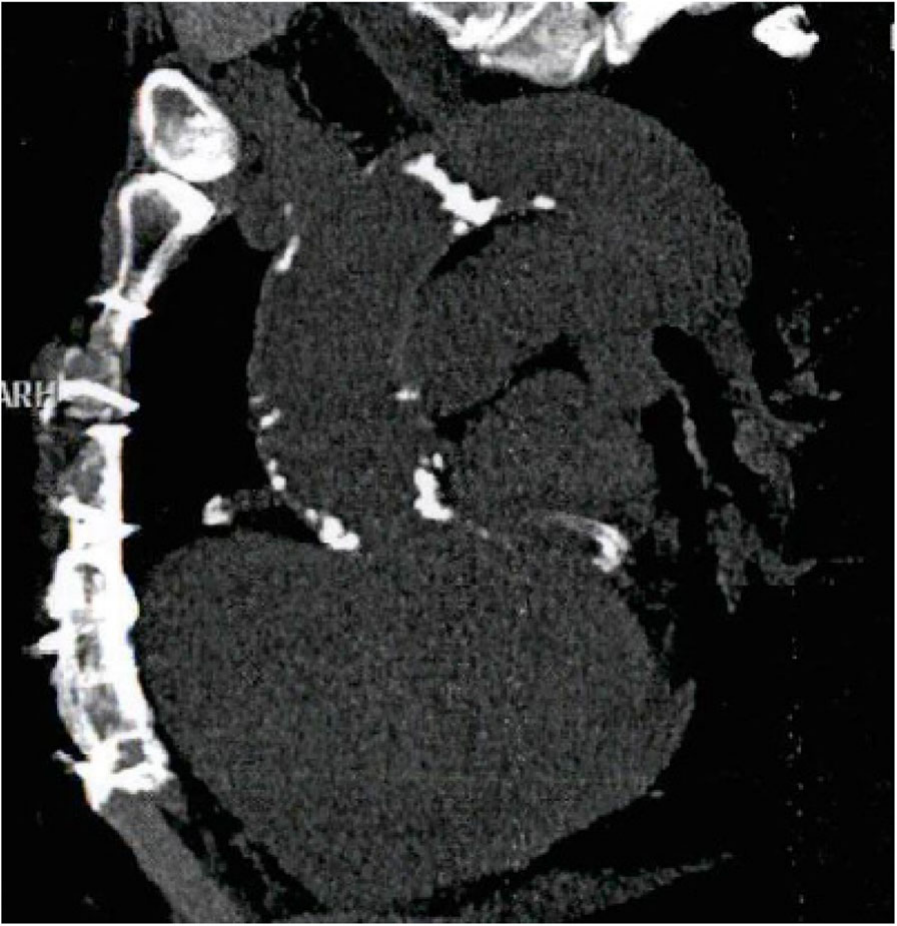
Preoperative computed tomography scan of the chest showing calcific root, ascending aorta and arch

**Fig. 2:**
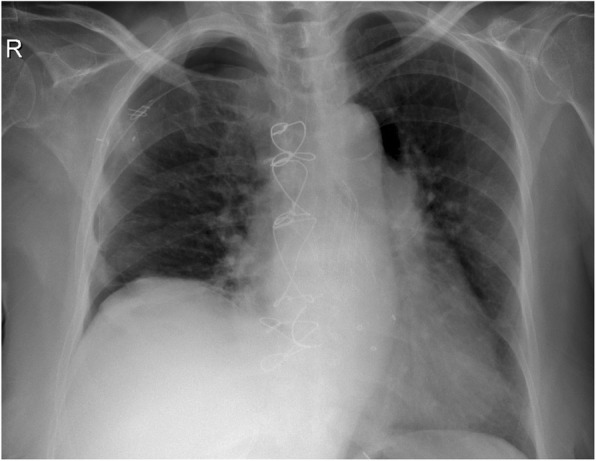
Pre-discharge chest X-ray showing the implanted aortic valve, bioprosthetic mitral valve, and tricuspid valve ring

A female patient aged 64 years old presented with chest pain and progressive dyspnea (New York Heart Association Class II). The patient had Ross operation 40 years ago for rheumatic aortic valve disease. Twenty years after the Ross procedure, the patient had aortic valve replacement with a tissue valve and open mitral commissurotomy for concomitant mitral valve stenosis. The patient had concomitant coronary artery disease and stenting of the left anterior descending and the circumflex coronary arteries 2 years prior to the latest presentation. The patient had atrial fibrillation, and the echocardiography showed mild global hypokinesia of the left ventricle, severe mitral and tricuspid regurgitation, severe mitral stenosis with the valve area of 1.1 cm^2^, calcific aortic tissue valve with moderate aortic stenosis, and severe aortic regurgitation. The ejection fraction was 45%, and pulmonary artery systolic pressure was 50 mmHg. The chest computed tomography scan showed calcific ascending aorta (Fig. 1), and the carotid Doppler showed 50% bilateral stenosis. After a multidisciplinary discussion, we decided to implant a transcatheter aortic valve and perform open surgery to replace the mitral and repair the tricuspid valve. The aortic valve was replaced with Evolut R 29 mm (Medtronic Inc., Minneapolis, Minnesota, USA). After 3 months, the patient had mitral valve replacement through a median sternotomy. There was a 1 cm distance between the Evolut R valve and the brachiocephalic artery, and the aorta was heavily calcific, making the aortic cross-clamp not feasible. We decided to perform beating mitral valve replacement under hypothermia (30 °C) with no aortic cross-clamp nor cardioplegic arrest. The aorta was cannulated just below the brachiocephalic artery, and both superior and inferior vena cava were directly cannulated. Transeptal approach was used, and the mitral valve was replaced with Mosaic tissue valve 25 mm (Medtronic Inc., Minneapolis, Minnesota, USA), and the tricuspid valve was repaired with a Tri-Ad semi-rigid ring 26 mm (Medtronic Inc., Minneapolis, Minnesota, USA). The insertion of an aortic root vent was not feasible because of the hostile aorta. The patient had right ventricular dysfunction with difficult weaning from cardiopulmonary bypass, and she required extracorporeal membrane oxygenation (ECMO) support, intra-aortic balloon pump (IABP), and high inotropic support, and the course was complicated by pleural effusion requiring drain insertion (Fig. 2). The ventricular function recovered after 1 day of ECMO support, and the patient was weaned from mechanical support gradually. Pre-discharge echocardiography showed 9 mmHg pressure gradient on the aortic valve with a mild paravalvular leak, functioning prosthetic mitral valve, and trivial tricuspid regurgitation. The hospital stay was 33 days.

## Discussion

The optimal valve prosthesis is still a subject of ongoing researches, and several factors govern the choice of the valve prosthesis for each patient. The Ross procedure has the advantages of growth potential and avoiding the use of anticoagulants, and compared to other prostheses, the Ross procedure was associated with better survival and freedom from valve-related complications when used in young- and middle-aged patients [1]. However, the long-term results are not optimal in rheumatic patients, and those patients require reoperation for autograft dysfunction or mitral valve lesions [2].

Despite the expansion of TAVI indications to include low-risk patients, technical challenges hinder the use of this technique in all patients, and the risk of future reinterventions will be an issue. Concomitant mitral valve prosthesis increased the complications after TAVI [5], and it was found that mitral stenosis increased the incidence of post-TAVI paravalvular leak and all-cause mortality [4].

We presented a case that had the Ross procedure 40 years ago for rheumatic aortic valve disease, and the patient had valve-in-valve TAVI and beating mitral and tricuspid valve surgery. TAVI in patients with prior Ross was rarely reported in the literature [6]. Additionally, the patient had prior aortic valve replacement with a tissue valve, which presents another challenge for TAVI with the possibility of increased postoperative pressure gradient and paravalvular leak. The postoperative maximum pressure gradient on the aortic valve was 9 mmHg, and the patient had a mild para-aortic leak. The degree of paravalvular regurgitation did not increase after mitral valve replacement.

Our patient had heavily calcific aorta, and surgical aortic valve replacement or the aortic-cross clamp would be risky. The risk of air embolism increases with beating mitral valve surgery, [7] and measures to protect this patient from the risk of air embolism were not feasible because of the hostile aorta. The use of aortic cross-clamp or aortic root vent was not possible; additionally, it may dislodge calcium from the aortic wall and cause systemic embolization with more extensive damage. We believe that the right ventricular dysfunction that occurred after mitral valve replacement was due to air embolism of the right coronary artery. The patient could not be weaned from cardiopulmonary bypass and required mechanical circulatory support. The ventricular function recovered gradually, and the patient was weaned from the circulatory support. Despite the increased risk of air embolism after beating mitral valve surgery, the patient had no clinical or radiological evidence of stroke.

The patient had concomitant coronary artery disease and preoperative mild global hypokinesia of the left ventricle, which could be a risk factor for difficult weaning from cardiopulmonary bypass. However, in-stent thrombosis was a remote possibility since the patient was heparinized and coronary perfusion was maintained throughout the procedure.

## Conclusion

Surgical MVR is feasible after TAVI and did not distort the implanted aortic valve or increase the para-valvular leak. Additionally, Ross patients can benefit from TAVI, which decreases the risk of open surgery, especially in those with hostile autograft.

## Data Availability

Available upon request

## Abbreviations

CT: Computed tomography
ECMO: Extracorporeal membrane oxygenation
IABP: Intra-aortic balloon pump
MVR: Mitral valve replacement
TAVI: Transcatheter aortic valve replacement
